# New Capabilities of the MuscleX Data Reduction Package for Fiber Diffraction from Muscle and other Fibrous Systems.

**DOI:** 10.1063/4.0001048

**Published:** 2025-10-27

**Authors:** Thomas C Irving

**Affiliations:** Illinois Insitute Of Technology, Chicago, Illinois, USA

## Abstract

For many years, analysis of fiber diffraction patterns from muscle was a rate limiting step in the preparation of experimental results for publication. Most analysis procedures made extensive use of FIT2D supplemented by locally developed or commercial software tools which were not specifically designed for the task. Analyzing the data from a single experiment could take many weeks or months. The open source "MuscleX” data reduction suite was written to address this bottleneck. While many of its routines have been heavily optimized for analysis of small-angle X- ray patterns from Muscle, e.g. The “Equator’ routine can analyze the equatorial patterns from the hexagonally packed myofilaments in the sarcomeres of striated muscle, many of the routines are more general purpose, including the ’Quadrant-Fold” package averages the four quadrants of a diffraction pattern to improve signal to noise for more detailed analysis as well as perform global background as well as remove the lines in the patterns created by the dead zones between modules in common pixel array detectors. Other components of the suite include “Projection Traces” that automatically analyze the intensity and spacings of prominent reflections on the meridian and layer lines. In this presentation we will discuss recent enhancements to MuscleX that have expanded its capabilities and performance as well as discuss planned future developments.

